# Effect of *Satureja khuzistanica* essential oil (SKEO) extract on expression of *las*A and *las*B genes in *Pseudomonas aeruginosa*

**Published:** 2019-02

**Authors:** Davoud Iman Islamieh, Davoud Afshar, Davoud Esmaeili

**Affiliations:** 1 Department of Microbiology and Applied Microbiology Research Center, Systems Biology and Poisonings Institute and Applied Virology Research Center, Baqiyatallah University of Medical Sciences, Tehran, Iran; 2 Department of Microbiology and Virology, School of Medicine, Zanjan University of Medical Sciences, Zanjan, Iran

**Keywords:** *Pseudomonas aeruginosa*, *Satureja khuzistanica* essential oil, *las*A gene, *las*B gene

## Abstract

**Background and Objectives:**

Expressions of *las*A and *las*B genes of *Pseudomonas aeruginosa* are associated with bacterium pathogenicity. The present study was aimed to assess the effect of *Satureja khuzistanica* essential oil (SKEO) extract on expression of *las*A and *las*B genes in *P. aeruginosa*.

**Materials and Methods:**

*Pseudomonas aeruginosa* isolates were cultured in Mueller Hinton broth containing sub-inhibitory concentrations of SKEO and total RNA extracted using Trizol method. cDNA was synthesized using random Hexamer primer and finally the expression of *las*A and *las*B genes carried out by real-time PCR.

**Results:**

The MICs of SKEO extract for PA9, PA10, PA11, PA13, PA41 and PA42 isolates were 8, 8, 8, 9, 7 and 12 μg/ml, respectively. Statistical analysis for 6 isolates revealed that the reduction in expression of *las*A and *las*B genes under SKEO treatment was significant (P<0.05).

**Conclusion:**

The insignificantly increasing of *las*B gene expression may lead to low virulent strains, for probably reason that the strain's exotoxin A are destroyed in the high amount of protease. In conclusion, using of SKEO in burned patients infected with *P. aeruginosa* may be effective; however, it is better to assess the spectrum activity of SKEO, pharmacokinetics, potency and its toxicity in human cells.

## INTRODUCTION

*Pseudomonas aeruginosa* is a Gram negative pathogen responsible for nosocomial and opportunistic infections, which comebacks to organism's intrinsic resistance to many antibiotics and its virulence factors. There are many mechanisms, which causes bacterium to resist to antibiotics such as, expression of β-lactamases, antibiotic inactivating enzymes and over-expression of efflux pumps (1).

In healthy individuals, most of the infections that are related to *P. aeruginosa* are opportunistic rather than primary diseases.

Cystic fibrosis (CF) is one of the opportunistic infections with high prevalence and in most cases is associated with *P. aeruginosa* (2, 3), although multiple bacterial species could also involve in progressing of CF associated infection (4).

Hospital-acquired infections known as nosocomial infections are infections acquired by patient during hospitalization. These infections are caused by several organisms and *P. aeruginosa* is one of the commonly isolated organisms (5). Nosocomial infections are commonly occurred in many countries, which comeback to their low and limited hygiene level (6). Existing of multidrug resistance (MDR) bacteria such as *P. aeruginosa* has been made it as a new challenge to organize a successful plan to eradicate nosocomial infections. Recently, most of strains isolated from hospitalized patients have MDR phenotype and antibiotic resistance is also increasing among isolates (7). This subject compromise the efficacy of antimicrobial therapy and effort to find new antimicrobial agents.

*P. aeruginosa* has a more complicated pathogenesis, which comebacks to its several virulence genes involved in its pathogenicity. A number of virulence genes such *astox*A, *exo*S, *las*A and *las*B have been identified in virulent strains (8). The *las*A and *las*B genes encode elastase enzymes, which have proteolytic activity and cause lung tissue and skin damages. Hence, the expression of these genes associated with species pathogenicity, especially in skin-burned patients (9).

*Satureja khuzistanica* is one of the native plants in Iran and its extract, *Satureja khuzistanica* essential oil (SKEO) traditionally used to treat patients with various diseases (10, 11).

The present study was aimed to assess the effect of SKEO extract on expression of *las*A and *las*B genes in MDR strains of *P. aeruginosa*.

## MATERIALS AND METHODS

### Preparation of plant.

Aerial parts of *S. khuzistanica* plant were collected from Khorramabad of Iran during summer of 2013 and recognition and categorization of the plant substance performed by Barij Essence Company (Iran). The essential oil of plant was gained by steam distillation of plant and then dried with sodium sulphate at 4°C (12).

### Bacterial isolates.

A total of 6 *P. aeruginosa* isolates with multidrug resistance phenotype (MDR) were cultured on MHA medium and incubated at 37°C for 24 h.

### Determination of MIC of SKEO oil.

SKEO oil was dissolved in dimethyl sulfoxide (DMSO) with a final concentration of 0.1 mg/mL as stocke and stored at 4°C away from heat and direct light. For each isolate, the volumes of 100, 120, 140, 160, 180, 200, 220 and 240 μl of SKEO (0.1 mg/mL) were added into eight sterile glass tubes (13 by 100 mm), reached on 2 ml by MHB containing fresh cultures (5×10^5^ CFU/ml) and were finally incubated for 18–24 hours at 37°C. About 10 μl from incubated tubes were transferred on MHA and incubated for 18–24 hours at 37°C. All tests were done in duplicate and positive and negative controls were also applied with bacteria and without oil, respectively.

### RNA extraction.

Following determination of MIC, isolates were cultured on LB broth with SKEO (at lower than MIC level) and incubated at 37°C for 24 h. Extraction of total RNA was carried out using commercial RNA extraction kit (Trizol Reagent; BRL Life Technologies), electrophoresed in 1% gel agarose and visualized under UV light of gel documentation system (Bio-Rad, Germany). Concentration of the extracted RNA was measured at wavelength 260 nm using Nanodrop spectrophotometer ((Nano Drop, Wilmington, DE, USA). To remove genomic DNA contamination, all samples were treated with DNase-I (1U /1 μg RNA) and kept at 37°C for 0.5 h. Lack of residual DNA was verified by PCR using RNA as a template (13).

### cDNA synthesis.

Synthesis of cDNA was carried out in a total volume of 20 μl reaction mixture including 2 μg of extracted RNA, 1 μl of dNTPs (10 mM), 50 ng of random Hexamer primer, 2 μl of M-MuLV buffer (10X) and 100 U of M-MuLV reverse transcriptase under an initial denaturation at 65°C for 5 min and reverse transcription steps at 42°C for 1 h (Vivantis, Malaysia). The products were immediately applied to RT-PCR and Real-time PCR assays.

### RT-PCR and Real-time PCR.

The RT-PCR reaction was performed using Revert Aid first strand cDNA synthesis kit (Fermentas, USA) in a total volume of 25 μl reaction mixture including 1 μg of cDNA, (10 mM), and 0.3 pmol of each primer (Table 1). PCR condition included an initial denaturation at 95°C for 2 min, 35 cycles of 95°C for 30s, 57°C for 30s and 72°C for 30s and a final extension at 72°C for 5 min. For internal control, *gyr*A was used. PCR products were electrophoresis in 1% agarose gel and visualized under UV light of gel documentation system (Bio-Rad, Germany).

**Table 1.** Oligonucleotides used in study.

**Primer**	**Sequence**	**Amplicon size (bp)**
*las*A-F	TGCATTTCTCGCTGCTCTAC	107
*las*A-R	GCGACAGTCGTTGTCGTAGT	
*las*B-F	AGTTTGGACACGTCGATCAG	78
*las*B-R	GCTTGACCTGTTGTTCGTTG	
*gyr*A-F	GGTCTGGGCATAGAGGTTGT	121
*gyr*A-R	GAAGATCGAGGGTATTTCCG	

Real-time PCR was also carried out using Corbett Thermocycler model gradient Palm Cycler under following conditions: 12.5 μl of Maxima SYBR Green/ ROX qPCR Master Mix 2X (Fermentas, USA), 2 μl of templates and 0.3 pmol of each primer (Table 1). The relative calculation of the gene expression according to the Livak method (ΔΔCt method), also known as 2^−ΔΔCt^ or comparative Ct method, is performed without modifying its efficacy, and it is assumed that the desired doubling of target and reference DNA genes runs throughout each cycle. In this study, the results of Real-time PCR were analyzed by SPSS software and ANOVA test (14).

## RESULTS

### MIC of SKEO.

For each isolate, the amount of 4, 5, 6, 7, 8, 9, 10, 11 and 12 μg/ml of SKEOwere examined and their MIC results are shown in Table 2.

**Table 2.** MICs of SKEO against *P. aeruginosa* isolates

***P. aeruginosa* isolates**	**MIC (μg/ml) SKEO**
PA9	8
PA10	8
PA11	8
PA13	9
PA41	7
PA42	12

### Effects of SKEO oil on *las*A and *las*B genes expression.

Relative-quantitative gene expression levels of *las*A and *las*B genes were calculated to comparing with control gene (*gyr*A). Melting curves of the target genes showed that there were any non-specific amplicons or primer-dimers.

The result analysis by SPSS version 22 demonstrated relation gene expression before and after treatment is meaningful (P<0.05).

Statistical analysis for 6 isolates revealed the decline in expression of *las*A and *las*B genes under SKEO treatment was significant (P<0.05). However, at the different concentrations of SKEO, the expression rate of *las*A and *las*B genes were found to be insignificant (P>0.05).

## DISCUSSION

Recently, the antimicrobial activity of *S. khuzistanica* has been shown to be associated more with its two important components: thymol and carvacrol (15, 16). In the present study, the effects of its extract (SKEO) on the expression of *P. aeruginosa las*A and *las*B genes were evaluated. These genes encode elastase enzymes, which play an important role in the pathogenicity of *P. aeruginosa* in burned patients (17).

As shown in the result paragraph, the MICs values of SKEO extract were partly similar among isolates, indicating a homogenous susceptibility against extract. The activity of herb extracts against bacteria has been previously shown. A study reported that in comparison with the Gram-positive bacteria, the *Pseudomonas* spp. were susceptible towards herb extracts (18).

In our study, while the SKEO extract decreased the expression of the *las*A, the *las*B gene expression increased, insignificantly. Huerta et al. showed that some of herb extracts decreasing pyocyanin formation also reduced the expression and activity of elastase (19). This property indicates that the herb extracts may affect some factors that are commonly involved in the expression of pyocyanin synthesizing enzyme and elastase coding genes.

The results showed that the expression of *las*B gene (P>0.05) increased in different concentrations of SKEO, Insignificantly. It has been determined that the strains with high amount protease are less virulent and the strains with low amount protease cause more severe clinical symptoms. Destroying of exotoxin A by strains protease may be close to reality, although it may have many causes behind the production of protease and virulence capacity (20).

*Pseudomonas aeruginosa* has an enzyme called elastase, which is coded by *las*A and *las*B genes. This enzyme has the ability to decompose cytokines, leukocytes and defense cells, and also plays a role in the decomposition of lung and vascular elastin tissues. *Ecthyma gangrenosum* (EG) is a well-recognized but uncommon cutaneous infection classically associated with *P. aeruginosa* bacteremia. If it can be controlled gene virulence by the drug substance, it is possible to reduce the bacterial pathogenicity. This research demonstrated that *S. khuzistanica* can reduce the expression of *las* gene.

It seems that using of SKEO in burned patients infected with *P. aeruginosa* can be effective; however, it is better to assess the spectrum of activity, pharmacokinetics, potency and its toxicity in human cells and animal model.
